# Evictions and short-term all-cause mortality: a 3-year follow-up study of a middle-aged Swedish population

**DOI:** 10.1007/s00038-016-0931-8

**Published:** 2016-12-09

**Authors:** Yerko Rojas

**Affiliations:** Stockholm University, Stockholm, Sweden

**Keywords:** Eviction, Ill-health, Housing, Home, Mortality, Sweden

## Abstract

**Objectives:**

This study sets out to explore whether being forcibly removed from one’s home is related to all-cause mortality.

**Methods:**

With the help of unique register data covering all middle-aged persons registered at the Swedish Enforcement Authority with a case closed by an eviction during the period 2009–2011 (*n* = 2092), evictees’ deaths from any cause that occurred within 3 years of the date of the eviction are compared with the all-cause mortality of a random sample of the Swedish population (*n* = 426,117). The analysis is based on penalized maximum likelihood logistic regressions.

**Results:**

Those who had been evicted from their homes were found to be approximately one and a half times more likely to die from any cause than those who had not been exposed to this experience (OR = 1.59), controlling for several demographic, socio-economic and health conditions prior to the date of the eviction.

**Conclusions:**

The results provide support for the notion that the experience of losing one’s dwelling place should be treated as a major life event in its own right, just like other well-established social stressors.

## Introduction

The importance of place, and particularly the significance of the dwelling place, has been well established in the social philosophical literature (Fox 2006). A dwelling is more than just bricks and mortar, it is a source of identity and meaningfulness, involving a sense of security (usually referred to as ontological security) (Fox 2006; Somerville 1992). This inseparability of persons from the places they inhabit has been argued to be so essential that its most extreme manifestation may involve illness and death resulting from the forcible removal of a person from his/her dwelling place (Fox 2006; Malpas 1999). However, although a number of small-sample Swedish studies among selected groups of evicted people have suggested a heightened risk for all-cause mortality within this group, the degree to which this increased risk can be attributed to the actual loss of the dwelling remains largely unanswered, at least from an empirical point of view (cf. Eriksson et al. 2010; Stenberg 1990).

In fact, despite the prevalence of eviction (millions of households across the world are evicted every year), “we know next to nothing about its impact on people’s lives” (Desmond and Kimbro 2015). Part of the explanation for this lack of knowledge is that the differential impact of a home eviction can only be assessed if we are able to consider the conditions that lead to the actual eviction in the first place, which has to date proven very difficult, since there are substantial difficulties associated with locating and reaching larger groups of individuals who have been subjected to this form of severe corrective sanction on the part of society (Eriksson et al. 2010). Furthermore, and no less importantly, evictions have until recently been neglected as a social problem by social scientists and policy makers alike (Desmond and Kimbro 2015; Stenberg et al. 2011).

In an attempt to deepen our understanding of the differential impact of home eviction on all-cause mortality, this study makes use of a unique opportunity, offered by the Microdata Online Access (MONA) system at Statistics Sweden, to follow all middle-aged women and men who were registered at the Swedish Enforcement Authority with a case closed by an eviction between 2009 and 2011 for a 3-year period using nationwide register data.

### Major life events and all-cause mortality—the role of home evictions

The most common operational meaning of stress is a “life change event”, that is, a “discrete and observable event representing change and thus requiring some social and/or psychological adjustment on the part of the individual” (Wheaton et al. 2013). Losing one’s job, getting a divorce, being assaulted or robbed and the like are all typical types of life change events that have interested social scientists exploring the impact of social stressors on health (Thoits 2010; Wheaton et al. 2013). It is thought that the extensive behavioral adjustments (including adverse changes in health-related behaviors) that individuals make in response to a given major life event overtax their capacity to cope or adapt, leaving them more vulnerable to infection, injury or disease (Thoits 2010), and ultimately, to all-cause mortality (e.g., Roelfs et al. 2011).

If we accept that: (1) a dwelling is a “socio-spatial system” [that is, simultaneously and indivisibly a spatial and a social unit of interaction (Mallett 2004; Saunders and Williams 1988)]; (2) our identity is somewhat tied to location [that is, a dwelling is both “humanized and humanizing” (cf. Karjalainen 1993; Malpas 1999)]; and (3) a dwelling is a source of meaningfulness, involving a sense of security (Fox 2006; Somerville 1992), it is not difficult to arrive at the same conclusion as Desmond and Kimbro (2015), who in their recent study on the fallout of eviction argued that experiencing an involuntary loss of housing may result in a “scarring” akin to that which workers sometimes experience following the involuntary loss of their jobs, and that it may thus, in a similar way, also be considered an acutely stressful life event. Evictees themselves have described this experience as being “as if the plug had been pulled on their existence”, “as if the whole world came crashing down” (Flyghed and Stenberg 1993; Nilsson and Flyghed 2004).

It is on the basis of this understanding, in combination with the available evidence on the effect of major life change events, such as unemployment, on all-cause mortality (see Roelfs et al. 2011), that the current study hypothesizes that home evictions and death from any cause are detrimentally related to one another.

### Evictions in Sweden: a short background

The Swedish Enforcement Authority is the only agency in Sweden that is authorized to execute evictions. The main cause of home evictions is defaulting on rent payments. 85% of evictions have been estimated to be due to rent arrears. In approximately 5% of evictions, the main cause is causing a disturbance. The remainder is evicted due to the frequent late payment of rent (Socialstyrelsen 2010). There are no official statistics on home eviction. For Stockholm, however, it has been estimated that approximately 84% of officially reported evictions correspond to evictions from the domicile (SOU 2005:88), which, if applied to the whole country, would mean that approximately 1868 (0.84 × 2224) home evictions took place in Sweden last year (2015) (Kronofogden 2016).

Evictions are to a large extent a so-called urban phenomenon. Resource-weak groups, such as the unemployed and recipients of means-tested social welfare, have all been found to be considerably overrepresented among those who are evicted. This also holds true, but to a lesser extent, for individuals with a criminal record. For persons born abroad, the evidence is not as categorical, and suggests only a negligible overrepresentation. It has also been shown that men and unmarried persons are evicted to a greater extent than women and family households and that the mean age of those evicted is over 40 (Eriksson et al. 2010). Furthermore, suggestions for preventive work among evicted persons note the importance of tackling the problem of low levels of education within parts of this group (Socialstyrelsen 2008). Moreover, those who are evicted have been found to suffer from considerably more health problems than other tenants, being clearly overrepresented when it comes to in-patient care (Eriksson et al. 2010). It is worth noting that practically, all these factors are themselves also treated as risk factors for all-cause mortality in the literature (e.g., Nieuwbeerta and Piquero 2008; Roelfs et al. 2011; Sorlie et al. 1995).

### Aim of the study

This study sets out to explore whether the forcible removal of a middle-aged person from his/her dwelling is in itself related to all-cause mortality, even when controlling for well-established factors that may have led to the eviction in the first place.

## Method

### The study base—an evicted and a comparison group

The exposed group examined in this study includes all middle-aged, 30–65 years (at baseline), women and men who were registered at the Swedish Enforcement Authority (Kronofogden) with a case closed by a home eviction during the period 1 January 2009 to 31 December 2011. The data from the Swedish Enforcement Authority register have, with the help of Statistic Sweden, been linked to several other national registers. In this study, I make use of the linkages made with (1) the longitudinal integration database for health insurance and labor market studies (known in Sweden as LISA), the population statistics register, and the geography database; (2) the register of persons convicted of criminal offences; and (3) the National Patient Register and the National Cause of Death Register. These registers are administered by Statistics Sweden, the Swedish National Council for Crime Prevention and the National Board of Health and Welfare, respectively.

The comparison group consists of all middle-aged women and men of a 10% random sample of the Swedish population, drawn on December 31, 2008 by Statistics Sweden. The data set for the comparison group contains the same information from the national registers as the data set for the individuals from the Swedish Enforcement Authority’s register. The individuals in the evicted group were removed from the sample population before the comparison group was produced.

### Analytical strategy

The available information on cause of death for those evicted in 2011 is limited to the years 2011–2014, that is, to a follow-up period of 3 years. As a consequence of this, the analysis of the evicted population is restricted to the all-cause mortality occurring within 3 years of an approximate eviction date at any point during the period 2009–2011. In addition, the comparison with the random sample of the Swedish population is restricted to a 3-year follow-up period, more precisely, to death from any cause occurring within 3 years after the sample was drawn (from January 1, 2009). The control variables are measured at baseline for both the evicted and the comparison group, that is, during the calendar year preceding the start of the follow-up period.

The analysis focuses only on the date of the first eviction executed during the observation period. It is extremely rare for an individual to be evicted more than once in the course of a short period of time, since following an eviction, it is very difficult to obtain a new dwelling from which one can then be formally evicted again (Eriksson et al. 2010). Furthermore, personal identification numbers that were identified as erroneous have also been excluded, as cases of emigration occurred during the study period (emigration could only be specified for the period to December 2013). Moreover, the analysis only considers persons for whom complete data are available on all the variables included in the models.

The relationship between the independent/control variables and all-cause mortality has been estimated using penalized maximum likelihood logistic regression (firthlogit). Firthlogit is a technique that is suited to dealing with situations in which the event of interest is rare (cf. Rojas and Stenberg 2016), which is the case here in the sense that the proportion of deaths from any cause in the data is lower than 5% (cf. King and Zeng 2001). Firthlogit is available as a subroutine in STATA (Firth 1995; Hilbe 2009; StataCorp 2015).

### Dependent variable

All-cause mortality is defined as death from any cause registered in the Swedish Cause of Death Register at any point during the follow-up period.

### Independent variable

Being forcibly removed from one’s home is defined in terms of the date on which the Swedish Enforcement Authority registered that the application for eviction submitted by the landlord had been closed due to eviction. This is usually registered by the enforcement officer on the same day as the eviction takes place, but registration may also occur one or a few days later.

### Control variables

The control variables have been measured in accordance with the analytical strategy described above, and are defined as follows. *Large city* living in one of the following three municipality types: metropolitan, suburban, or larger cities. *Unemployment* being registered as unemployed at the relevant authorities for at least one day over the course of a 1-year period. *Criminal record* having been found guilty of a criminal offence at least once over a 1-year period. *Foreign born* having been born outside Sweden. *Family constellation:* single persons/all other family constellations (married families [including civil unions], cohabiting families [with common children], and one-parent families). *Age* year of birth. *Gender* women/men. *Education* pre-upper secondary, upper-secondary and post-upper-secondary education. *Social welfare recipiency* having received means-tested social assistance at least once over the course of a year. Finally, *Ill*-*health* is measured using three different indicators: (1) having been recorded in the Swedish in-patient care register for any cause at least once over a 1-year period and (2) having received sickness cash benefit from the Swedish Social Insurance Agency at least once (measured in terms of net days) over a 1-year period. The principal requisite for being eligible for this benefit is that the person has an illness or injury that reduces his/her ability to work by at least 25%. To be entitled to this benefit, one has to be 16 years or older and have had a minimum annual income from work (in 2010, the qualifying income for sickness cash benefit was approximately 10,200 SEK (1020 EUR) (Mulder, 2011); and (3) having received sickness compensation from the Swedish Social Insurance Agency at least once over the course of a year. Sickness compensation is granted to persons aged 30–64 with a long term or permanently reduced work capacity (by at least 25%). It can be granted to persons with zero earnings. Since July 2008, this benefit is only granted to those with a permanently reduced work capacity (Lidwall 2013);

## Results

The study base is composed of an evicted and a comparison group, comprising a total of 2,092 and 426,117 individuals, respectively (see Table 1). A total of 4012 deaths from any cause are included in the analysis, of which 73 occurred in the evicted group and 3939 in the comparison group (see Table 1). The proportion that experienced all-cause mortality within the evicted group is approximately four times as large as the corresponding proportion within the comparison group (see Table 1). Overall, the distributions of the control variables differ considerably between the evicted group and the control group, although not to the same extent as the level of all-cause mortality (with the exception of unemployment, social welfare recipiency, criminality, and in-patient care), confirming the marginalized condition of those who are forcibly removed from their homes (see Table 1).

**Table 1:** Distributions of dependent and control variables included in the models (Sweden, 2009–2014)

Variables	Respondents	
	Evicted group (*n* = 2092)	Comparison group (*n* = 426,117)
Death from any cause% (*N*)	3.5 (73)	0.9 (3939)
Age		
Age at baseline M (SD)	45.0 (9.3)	47.6 (10.3)
Gender		
Men % (*N*)	64.1 (1341)	50.4 (214,563)
Place of birth		
Foreign born % (*N*)	24.5 (513)	15.7 (67,030)
Unemployment		
Unemployed % (*N*)	37.1 (776)	7.1 (30,359)
Education		
Pre-upper-secondary % (*N*)	32.1 (672)	16.3 (69,372)
Upper-secondary % (*N*)	57.0 (1193)	47.6 (202,786)
Place of residence		
Living in a large city % (*N*)	54.0 (1130)	62.6 (266,678)
Family constellation		
Single % (*N*)	66.6 (1394)	28.3 (120,634)
Criminality		
Convicted of a criminal offence % (*N*)	17.7 (370)	1.1 (4771)
Social welfare recipiency		
Received social assistance % (*N*)	49.4 (1033)	3.5 (14,913)
Ill-health		
Received in-patient care % (*N*)	11.2 (235)	1.0 (4116)
Received sickness cash benefit % (*N*)	13.5 (283)	10.8 (46,051)
Received sickness compensation % (*N*)	21.8 (456)	12.0 (51,302)

The results from the penalized maximum likelihood logistic regression analysis are presented in Table 2. In Model 1, we see that forcibly removing an individual from his/her home is significantly related to all-cause mortality, with a corrected OR of 3.90. In other words, those who had been evicted from their homes were approximately four times as likely to die from any cause as those who had not been exposed to this experience. As can be seen in Model 2, this relationship is somewhat weakened when adjusted for age, gender, place of birth, family constellation, and place of residence (cf. OR = 3.90 with OR = 3.60). However, the effect of eviction does not vary with any of the newly introduced variables; none of the multiplicative terms used to check for possible interaction effects were statistically significant.

**Table 2:** Penalized maximum likelihood logistic regression of home eviction and short-term all-cause mortality among 30–65 years in Sweden 2009–2014

Variable	Model 1 Crude OR (95% CI) (*p*)	Model 2 Adjusted OR (95% CI) (*p*)	Model 3 Adjusted OR (95% CI) (*p*)	Model 4 Adjusted OR (95% CI) (*p*)
Home eviction				
Forcibly removed from the dwelling (reference category: Other)	3.90 (3.08–4.93) (0.000) *	3.60 (2.83–4.58) (0.000)*	2.33 (1.81–3.00) (0.000)*	1.59 (1.23–2.07) (0.000)*
Age				
Age at baseline (continuous)		1.10 (1.09–1.10) (0.000)*	1.09 (1.08–1.09) (0.000)*	1.09 (1.08–1.09) (0.000)*
Gender				
Men (reference category: women)		1.58 (1.48–1.69) (0.000)*	1.85 (1.73–1.97) (0.000)*	1.77 (1.66–1.89) (0.000)*
Place of birth				
Foreign born (reference category: born in Sweden)		1.05 (0.96–1.15) (0.258)	0.94 (0.86–1.03) (0.164)	0.87 (0.79–0.96) (0.004)*
Family constellation				
Single (reference category: other family constellations)		2.10 (1.97–2.24) (0.000)*	1.72 (1.61–1.84) (0.000)*	1.67 (1.56–1.78) (0.000)*
Place of residence				
Living in a large city (reference category: other)		0.93 (0.88–0.996) (0.037)*	0.97 (0.91–1.03) (0.342)	1.00 (0.94–1.07) (0.886)
Ill-health				
Received in-patient care (reference category: other)			4.38 (3.87–4.96) (0.000)*	3.80 (3.35–4.31) (0.000)*
Received sickness cash benefit (reference category: other)			2.47 (2.28–2.66) (0.000)*	2.49 (2.30–2.69) (0.000)*
Received sickness compensation (reference category: other)			3.07 (2.86–3.28) (0.000)*	2.88 (2.68–3.09) (0.000)*
Unemployment				
Unemployed (reference category: other)				0.99 (0.87–1.13) (0.861)
Education				
Pre-upper-secondary (reference category: post-upper-secondary education)				1.52 (1.39–1.67) (0.000)*
Upper-secondary (reference category: post-upper-secondary education)				1.24 (1.14–1.35) (0.000)*
Criminality				
Convicted of a criminal offence (reference category: other)				1.55 (1.28–1.87) (0.000)*
Social welfare recipiency				
Received social assistance (reference category: other)				1.93 (1.70–2.20) (0.000)*
Death from any cause	4012	4012	4012	4012
Total study population (*n*)	*n* = 428,209	*n* = 428,209	*n* = 428,209	*n* = 428,209

Crude and adjusted odds ratios (OR) with 95% confidence intervals (CIs) and *p* values (*p*)

* Statistically significant (at the 5% level)

When ill-health (measured using three different indicators) is included in the analysis, the effect of eviction on all-cause mortality remains significant but decreases in strength, from an OR of 3.60 to one of 2.33 (see Model 3). Once again, the use of multiplicative terms to check for interactions between the new control variables and home eviction showed no significant interaction effects.

In the final model, four additional control variables are introduced into the analysis (social welfare recipiency, unemployment, education, and criminality). The effect of eviction decreases once again, culminating in an odds ratio of 1.59, but continues to remain significant. In this model too, no statistically significant interaction effects were found between being forcibly removed from the home and the newly introduced controls.

In Table 3, the final model is replicated excluding, one at a time, the three leading causes of death found among the evictees. The objective here is to examine how sensitive the final results are to specific causes of death. The effect of eviction remains practically the same, both in terms of size and statistical significance (cf., Table 3 with Model 4 in Table 2).

**Table 3:** Penalized maximum likelihood logistic regression of home eviction and short-term all-cause mortality among 30–65 years in Sweden 2009–2014, excluding circulatory disease (I00–I99) neoplasms (C00–D48) and external causes (V01–Y98) of death, respectively

Variable	Sensitivity test 1 (death from all causes except circulatory disease) Adjusted OR (95% CI) (*p*)	Sensitivity test 2 (death from all causes except neoplasms) Adjusted OR (95% CI) (*p*)	Sensitivity test 3 (death from all causes except external causes) Adjusted OR (95% CI) (*p*)
Home eviction			
Forcibly removed from the dwelling (reference category: Other)	1.60 (1.19–2.14) (0.002)*	1.62 (1.21–2.17) (0.001)*	1.51 (1.12–2.04) (0.008)*
Age			
Age at baseline (continuous)	1.08 (1.08–1.09) (0.000)*	1.07 (1.07–1.08) (0.000)*	1.10 (1.10–1.11) (0.000)*
Gender			
Men (reference category: women)	1.55 (1.44–1.67) (0.000)*	2.43 (2.22–2.66) (0.000)*	1.67 (1.56–1.79) (0.000)*
Place of birth			
Foreign born (reference category: born in Sweden)	0.89 (0.80–0.98) (0.022)*	0.80 (0.71–0.90) (0.000)*	0.88 (0.80–0.97) (0.012)*
Family constellation			
Single (reference category: other family constellations)	1.66 (1.54–1.79) (0.000)*	1.97 (1.81–2.15) (0.000)*	1.61 (1.50–1.73) (0.000)*
Place of residence			
Living in a large city (reference category: other)	1.03 (0.96–1.11) (0.405)	0.91 (0.83–0.99) (0.024)*	1.03 (0.96–1.11) (0.363)
Ill-health			
Received in-patient care (reference category: other)	4.20 (3.66–4.84) (0.000)*	4.59 (3.97–5.31) (0.000)*	3.47 (3.01–3.99) (0.000)*
Received sickness cash benefit (reference category: other)	2.82 (2.59–3.07) (0.000)*	1.43 (1.26–1.61) (0.000)*	2.63 (2.43–2.86) (0.000)*
Received sickness compensation (reference category: other)	2.70 (2.50–2.92) (0.000)*	4.06 (3.71–4.45) (0.000)*	2.91 (2.71–3.13) (0.000)*
Unemployment			
Unemployed (reference category: other)	0.93 (0.80–1.08) (0.344)	1.29 (1.10–1.51) (0.002)*	0.94 (0.81–1.08) (0.371)
Education			
Pre-upper-secondary (reference category: post-upper-secondary education)	1.45 (1.31–1.61) (0.000)*	1.63 (1.44–1.85) (0.000)*	1.48 (1.34–1.63) (0.000)*
Upper-secondary (reference category: post-upper-secondary education)	1.19 (1.08–1.30) (0.000)*	1.31 (1.17–1.48) (0.000)*	1.20 (1.10–1.32) (0.000)*
Criminality			
Convicted of a criminal offence (reference category: other)	1.62 (1.31–2.00) (0.000)*	1.72 (1.39–2.12) (0.000)*	1.23 (0.98–1.56) (0.076)
Social welfare recipiency			
Received social assistance (reference category: other)	1.90 (1.64–2.21) (0.000)*	1.99 (1.70–2.32) (0.000)*	1.87 (1.62–2.16) (0.000)*
Death from any cause	3082	2296	3535
Total study population (*n*)	*n* = 426,078	*n* = 424,299	*n* = 427,227

Adjusted odds ratios (OR) with 95% confidence intervals (CIs) and *p* values (*p*)

International Classification of Disease, 10th Revision codes

* Statistically significant (at the 5% level)

## Discussion

Between the post-war period and the latest global financial crisis home evictions have been a hidden social problem (Hartman and Robinson 2003; Stenberg et al. 2011). An important part of the explanation for this, at least in Sweden, is that the steady increase in home evictions, since the mid-1960s has come to be explained as an unintended consequence of the provision of homes to poor households and families with social problems, i.e., to individuals who have a high eviction risk in the first place. Thus, the difficulties and consequences associated with home evictions have been viewed as being part of an ongoing process of social marginalization rather than as being attributable to the involuntary loss of the dwelling itself (cf. Eriksson et al. 2010; Stenberg et al. 1995, 2011). This notion of a “non housing-ready” population, which is also widespread in the current debate on pathways into housing (as an argument against the “housing first” model) (Tsemberis et al. 2004; Waegemakers Schiff and Schiff 2014) tends in other words to make the positive aspects of having a dwelling (or inversely, the negative aspects of losing it) conditional upon individuals ability to maintain their independent housing status. Thus, for those who are not members of the “housing-ready” population, the dwelling tends to be reduced to a mere question of bricks and mortar.

The results of this study challenge this view. In fact, the detrimental effect of eviction on all-cause mortality not only remains when controlling for a range of factors identified in the literature as being crucial to understanding the underlying reasons for home evictions, but also appears to be additive in nature. In other words, and in accordance with the assumptions outlined at the outset of this study, the results suggest that the dwelling plays an independent and fundamental role in relation to our wellbeing; that is, the negative aspects of forcibly losing one’ s dwelling, e.g., the loss of security [a need whose importance is comparable to the need for food and water (Kearns et al. 2000)], are not reducible to nor conditioned by the fact that one, prior to the eviction, had severe problems of various types.

Another important factor that has made it easy to ignore the view that a dwelling might be something more than just bricks and mortar for individuals involved in an eviction process is the argument that the concept of a “home” is ultimately an experiential phenomenon that is difficult to measure and articulate (Fox 2006). In fact, the lack of consensus on the importance of the dwelling has been one of the main obstacles to social workers employing all the legal means available to them to help those threatened by eviction at an early stage in the process and to thus preventing evictions from happening altogether (Kjellbom 2014).

While it is reasonable to accept that any attempt to “guess” the particular meaning of home for an individual occupier must be approached with caution, theoreticians in the field of housing studies have argued that this particular type of environmental intangible can both be identified and quantified if one focuses on the impact of losing it; that is, the importance of a home can be understood in negative terms, since the qualities associated with having a home typically emerge when the home is lost (Fox 2006). Hence, at least from a theoretical point of view, this study should be viewed as an indication that being forcibly removed from one’s dwelling constitutes a major life event with far-reaching detrimental consequences (cf. Desmond and Kimbro 2015; Fowler et al. 2015).

The current study is to the best of my knowledge the first to examine the relationship between home evictions and all-cause mortality using longitudinal, large-scale register data, and including an unprecedented variety of information, for an entire country (cf. Desmond and Kimbro 2015; Eriksson et al. 2010; Fowler et al. 2015). It is, therefore, important to be cautious about drawing wide-ranging conclusions on the basis of this single study. Having said this, the study’s findings are suggestive of two relatively straightforward policy implications.

First, the legitimacy of eviction needs, just like any other formal societal sanction, to be evaluated in relation to its consequences (including the unintended ones) (cf. Weiss 1998). Hitherto, home evictions have mainly been legitimized on the basis of their underlying intention, which is to promote the general payment moral in society (Eriksson et al. 2010). It is thought that “if people see that unpaid rents, installments or amortizations do not lead to any type of sanction, there is a risk that confidence in the common economy will disappear” (Westerberg 1999). Given the new information provided by the results of the current study, assessments of the legitimacy of the use of the evictions measure now need also to include a consideration of whether a heightened risk of death from any cause may be viewed as a proportionate outcome in relation to achieving the intention described above, particularly given that 80% of those evicted in Sweden have been found to have rent arrears of less than 2000 EUR (Eriksson et al. 2010). Second, the inclusionary and compensatory societal measures directed at minimizing the negative effects of this powerful corrective sanction also need to be re-evaluated in terms of their proportionality, given this newly acquired knowledge (Flyghed 2000).

### Limitations

Three main methodological considerations should be borne in mind when interpreting the results of this study. First, since the study is entirely based on register data, I lack self-reported information on confounders that might have influenced the results (Thygesen and Ersbøll 2014), e.g., self-reported leisure-time physical activity (Pekkanen et al. 1995). However, capturing representative and sizeable groups of evicted individuals by means of traditional surveys has to date proven to be very difficult (Eriksson et al. 2010), which makes register-based studies of the kind presented here extremely important (Thygesen and Ersbøll 2014).

Second, the analysis relies on a comparison with the general population as opposed to a population specified on the basis of being in possession of a dwelling. The reason for this is that there are no available register data in Sweden, covering the study period that would make it possible to identify this latter group. However, since one of the most important limitations associated with the use of the general population as a comparison group is the tendency to underestimate the real effect of a given exposure (which in this case would be the case if a large proportion of the comparison group was comprised of people living in insecure housing, e.g., renters without a lease), the use of this type of comparison group is common in this area of research even in cases, where it is possible to make this distinction, e.g., using the unemployed vs the employed population (cf. Roelfs et al. 2011).

Third, as the eviction date used in this study was inferred from the date on which the Swedish Enforcement Authority closed the enforcement case in question, the analysis only includes cases of home eviction that had no other enforcement matters pending at the time of the eviction. Hence, the validity of the study’s results is limited to this particular population of evictees. Having said this, the evicted population in Sweden is not characterized by having large or long-term debts at the agency (Flyghed 2000).

### Conclusion

Forcibly removing people from their homes has a detrimental impact on all-cause mortality. This effect is statistically independent of other important social stressors (e.g., unemployment), health conditions, and behavioral and demographic characteristics that are not only well-known risk factors for death from any cause, but also constitute the main risk factors for home eviction. Thus, the experience of losing one’s dwelling should be treated as a major life event in its own right; that is, it is neither reducible to nor conditioned by the factors that enable people to remain in possession of their homes. Having said this, further studies are needed to confirm these findings.
